# Supplementary material for: Hormonal responses to brief social interactions: The role of psychosocial stress and relationship status
Source: PLoS One. 2023 Jun 23;18(6):e0287153. doi: 10.1371/journal.pone.0287153 (PMC10289427; doi:10.1371/journal.pone.0287153)
Supplement: Attachment — Submitted filename: Response to Reviewers PONE-D-21-20363.docx [file pone.0287153.s002.docx]

Reference: PONE-D-21-20363

Title: Hormonal responses to brief social interactions: The role of psychosocial stress and relationship status

Journal: *PLoS ONE*

Dear Dr. Sokolov,

We would like to begin by thanking you and the reviewers for your time and thoughtful comments on our paper, which have contributed to an improved manuscript. We paid very close attention to the comments of the reviewers while preparing this revision and have documented the changes we have made below. In order to assist you in reviewing our manuscript’s revisions, we have included each of the reviewer’s comments in the decision letter below, and our response to a comment is italicized and underlined. We have also edited the manuscript to meet the journal requirements mentioned in the decision letter, including formatting for style requirements, editing the use of “male” and “female” as nouns, and clarifying data availability in our updated cover letter.

Thank you in advance for your review!

Sincerely,

Nora (Nickels) McLean

**Reviewer #1:**

1. The introduction is relatively lengthy, not necessarily because it is too long but because it presents a suit of detailed findings from other studies with no clear direction of where the authors go. The main theories regarding hormonal responsiveness to social interactions are not named and the main theories published are not cited. The authors should revise the introduction to expand on theory and merge the exposition of previous findings into less lengthy phrasing.
In the introduction the interplay between Testosterone and cortisol in their hormonal regulation and the negative feedback effects that cortisol has on testosterone should be presented and discussed and incorporated in the hypothesis.

*The authors have edited the introduction to expand on theory relevant to the research questions (e.g., the challenge hypothesis and the dual hormone hypothesis), as well as to help connect the expected fluctuations of these sex hormones to the research questions of interest. When possible, we have merged the exposition of previous findings to lead to less length phrasing. These recommendations have (very helpfully) led to a clearer flow between past findings and the current research questions.*

2. Line 47: please nominatively introduce the challenge hypothesis (Wingfield et al. 1990) and expand citation to other studies which directly cited it in non-human primates or other animals (e.g. Muller & Wrangham 2004; Hirschenhauser & Oliveira 2006; Girard-Buttoz et al. 2015).

*The introduction of testosterone in the manuscript has been edited to incorporate the conceptual framework of the challenge hypothesis and supporting nonhuman evidence.*

3. One key aspect of this hypothesis is differences in testosterone reactivity to social interactions depending on paternity status. It appears therefore crucial to control for this parameter in the analysis beyond simply assessing if individuals are single or not.

*In response to controlling for paternity and maternity status of men and women: only three participants reported having children at the time of the study; cortisol and testosterone levels did not significantly differ between those participants who had children and those who did not.*

4. Line 75: Here the authors point out the important effect of anticipation. However this parameter was not tested in their experiment since the participants did not know that they would be placed in a (albeit neutral) courtship opportunity situation. Such anticipatory principle is therefore not relevant to the current study.

*This is a helpful comment; participants were not aware of the social interaction that is going to happen. We have removed the anticipatory principle from this paragraph. A social interaction itself can involve stress during the experience, particularly in a potentially social evaluative scenario; therefore, the rest of this paragraph’s introduction to a stressful component to courtship remains in the manuscript.*

5. Line 93: Define ‘confederates’.

*The term “confederates” has been removed from description of this particular citation, and instead replaced with female “individuals”.*

6. Line 121: present what cortisol is and highlight that it regulates the hormonal stress response in mammals including humans. In general, beyond providing a list of what testosterone and cortisol do and have been found to do it would be useful to provide clear cut presentation of what they regulate (e.g. muscle mass, aggressiveness for testosterone and energy mobilization for cortisol) and what they are expected to be sensitive too and why.

*When introducing testosterone and cortisol, the authors have edited the manuscript to more thoroughly clarify the regulating functions of these hormones. This includes the clarification of important theories related to the two hormones, and better clarifying how their sensitivity may relate to our main and exploratory research questions.*

7. Line 123: What is presented in the following lines are not hypothesis but prediction and testable patterns… please formulate hypothesis first.

*The final paragraph of the introduction section has been rewritten to clarify hypotheses and predictions. Specifically, it refers to the main hypotheses and exploratory analyses that are explored in separate sections of the rewritten results section of the revised manuscript.*

8. The methods are overall clear and well detailed. As previously, mention the authors should use GLMM rather than anova to clarify methodology and include more control variables. The authors should also run first a full model with all the interactions considered and compared it to a null model using an ANOVA and then reduce this full model removing non-significant interactions to obtain a final reduced model where the interactions and single effects are interpretable. In fact, single effects in interactions cannot be interpreted on their own. As previously mentioned, the authors should control for some important factors such as the paternity status of the men and the maternity status of the woman as well as whether they failed or passed the mathematical test which may have played a role in their hormonal stress response (one can imagine that failing is more stressful and that individual who failed may have more struggle solving mathematical problems hence be exposed to a higher stress).

*Reviewers recommended two different statistical approaches for revisions for this manuscript. Based on concerns over power, the authors chose to split analyses and only investigate two-way interactions effects, focusing on specific research questions. Both the main research question and exploratory research questions were limited to two-way interaction effects to avoid concerns regarding test power, as recommended by Reviewer #2. The results section of the manuscript has been completely rewritten based on the new analysis.*

*Addressing paternity and maternity status of men and women: only three participants reported having children at the time of the study; cortisol and testosterone levels did not significantly differ between those participants who had children and those who did not.*

*Addressing performance on the mathematical test: the mathematical test that is completed as part of the Trier Social Stress Test is a serial subtraction task. No participant successfully completed the entire task by counting down to zero (with or without a mistake). Data was not collected on how many mistakes were made for each participant.*

9. Write men and woman rather than male and female, it should be clear right from the start that you test humans.

*Male and female are no longer used as nouns; they are replaced as adjective phrases or with men and women in the manuscript.*

10. Line 147: Clarify what is SES

*SES has been clarified as socioeconomic status in the manuscript.*

11. Line 217: Hormonal level is too general please indicate which level you mean… also the literature cited does not test and present clear result on circadian rhythm of cortisol and testosterone… please cite specific literature (e.g. for cortisol Doman et al. 1986).

*Cortisol and testosterone levels have been specified, and additional specific literature has been cited regarding diurnal rhythms and methodological procedures.*

12. Line 224: what concentration were the CV based on? Did you use high concentration and low concentration quality controls?

*Assay description in the method section of the manuscript has been edited to include specifics about both intra- and inter-assay CV calculations.*

13. Line 238: Provide citation and version for R and Jamovi. Can you specify what you sued R for?

*For the updated analyses, only jamovi was used. The citation and version have been added to the manuscript.*

14. Since the result section will be modified following the changes in statistical approach, I will not comment in details on this section. I would however recommend including a table of results to visualize which variables were included in the analyses, in particular in the final model. As presented it is hard to know if the approach is valid or not.

*Reviewers recommended two different statistical approaches for revisions for this manuscript. Based on concerns over power, the authors chose to split analyses and only investigate two-way interactions effects, focusing on specific research questions. Both main research questions and exploratory research questions were limited to two-way interaction effects to avoid concerns regarding test power, as recommended by Reviewer #2. The results section of the manuscript has been completely rewritten based on the new analysis.*

**Reviewer #2:**

My general impression is very positive. The manuscript is expertly written, the research questions are interesting, and the methods are appropriate. I really liked the limitations part in the discussion section (great that you discuss potential issues with salivary immunoassays).

This being said, I would like to share some thoughts that came to my mind while reading through the manuscript. The points raised do only address the results section and the rather complex analyses (that are sometimes hard to interpret). I really hope that my feedback is helpful and helps to improve the manuscript.

1. First, I am not sure whether I got this right, was the main analysis a 4-way interaction? Or did the authors compute several two-way interactions? This was not 100% clear to me, please specify what you did here in detail. For now, I assume an anova with a 4-way interaction (treatment group x sex x relationship status x saliva sample). If this is correct, I do have strong concerns regarding test power. Besides the fact that such higher order interactions are really complex and difficult to understand, three-way interactions require a much higher test power than two-way interactions (the problem is even more evident for four-way interactions). I would assume that these analyses are heavily underpowered and rather uninformative. Importantly, underpowered studies may not have the power to detect an expected (small) effect size, but, at the same time, underpowered studies that report significant effects might overestimate effect sizes which may lead to difficulties to replicate, due to a higher influence of random measurement error (Button et al., 2013). Here is my suggestion how to deal with this issue:
I would recommend splitting the analyses, only investigating two-way interaction effects. Focus on the separate research questions you want to answer. To me, it seems as if the two-way interaction between treatment group and saliva sample is more of a method validation (does the TSST lead to higher cortisol/ testosterone levels?) and might be the first step. Then think about the second step and whether it is relevant to e.g. include a treatment variable in the analyses of the next research question. There were planned exploratory analyses regarding singles vs. partnered participants. I would recommend to do them separately from the main analyses, as a second step. Ask yourself if, for answering this specific research question, is it relevant to distinguish between participants who were in the TSST vs. control condition here? Is it relevant to distinguish between male and female participants here, or could this be a next step?

*Reviewers recommended two different statistical approaches for revisions for this manuscript. Based on concerns over power, the authors chose to split analyses and only investigate two-way interactions effects, focusing on specific research questions. Both main research questions and exploratory research questions were limited to two-way interaction effects to avoid concerns regarding test power, as recommended by Reviewer #2. The results section of the manuscript has been completely rewritten based on the new analysis.*

2. Second, please report (standardized) effect sizes and interpret your results accordingly. Reporting standardized effect sizes would make it easy for the reader to compare effect sizes across different analyses here and across different studies in general (and might also be helpful for future meta-analyses). It would also make it possible for the authors and for each reader to think about the reported effects in terms of effect sizes, not only based on p-values.

*Standardized effect sizes and interpretations are now reported for all significant main and interaction effects reported in the manuscript.*

3. Page 17, line 252, something went wrong here (there are two p-values reported): “for control participants was not confirmed (t=1.24, p=1.0, p=0.002; Fig 1)”

*This error has been removed, due to new analyses replacing the prior results section of the original manuscript.*

4. I do understand that you are not allowed to openly share your data, but I want to encourage you to think about sharing your analysis scripts (e.g. R script or SPSS syntax) and maybe, if possible, some material used in this study. This would make it easier for interested scholars to replicate this study in the future or to evaluate the analyses. Every little step in the direction of open and reproducible science helps.

*.omt files (jamovi script files, written in R script) are being submitted with the revision. Separate files for main and exploratory analyses are prepared and have been submitted with the revision as a supporting information zip file.*
