# Supplementary material for: Hormonal responses to brief social interactions: The role of psychosocial stress and relationship status
Source: PLoS One. 2023 Jun 23;18(6):e0287153. doi: 10.1371/journal.pone.0287153 (PMC10289427; doi:10.1371/journal.pone.0287153)
Supplement: Attachment — Submitted filename: Response to Reviewers PONE-D-21-20363R1.docx [file pone.0287153.s003.docx]

Reference: PONE-D-21-20363R1

Title: Hormonal responses to brief social interactions: The role of psychosocial stress and relationship status

Journal: *PLoS ONE*

Dear Dr. Sokolov,

We would like to begin by thanking you and the reviewers for your time and thoughtful additional comments on our paper, which have once again contributed to an improved manuscript. We paid very close attention to the comments of the reviewers while preparing this second revision and have documented the changes we have made below. In order to assist you in reviewing our manuscript’s revisions, we have included each of the reviewer’s comments in the decision letter below, and our response to a comment is italicized and underlined.

Thank you in advance for your repeated review!

Sincerely,

Nora (Nickels) McLean

**Reviewer #1:**

In this revised version, the authors have made a great work at reformatting the introduction and have now a nicely introduced paper. The paper has improved overall and is now in a nice shape.

1. I have still a few comments and the authors should conduct an in depth detailed check of typos and bracket errors since I have seen a few and did not point each of them in detail.

*The authors have conducted an in-depth check for typos and have corrected typos that were not pointed out in detail by the reviewer’s comments.*

2. The method lacks a few details regarding the hormonal measurements and the choice of the time frame for sample collection.

*In the saliva sampling subsection of the method section, the authors have added information about the timing of saliva sample collection – both in terms of time of day and clearance delay, citing existing background citation used in the manuscript. In terms of hormonal measurements, edit have been made to the manuscript in the saliva sampling section as well (see edit #9 below for more detail).*

3. The term confederates should either be explained clearly or removed throughout.

*The term “confederates” has been removed throughout the manuscript; see detailed response below in change #5.*

4. Line 105: “consistent” not “consisten”.

*This typo has been resolved in the updated manuscript.*

5. Line 191: by ‘confederates’ do you mean ‘judges’? I thought you removed the term confederate throughout the manuscript, maybe this is a typo or has been forgotten here.

*In the first revision, the term “confederates” was removed when discussing a separate study previously performed. For this updated revision, the authors have removed the term “confederate” throughout the entire manuscript, clarifying that we are either referring to judges in the Trier Social Stress Test, or opposite-sex research assistants who assisted in the social interaction task.*

6. Line 210: same here and throughout method description, change confederate to another term or define it somewhere.

*The term “confederates” has been removed throughout the manuscript; see detailed response above in change #5.*

7. Line 230-232: Can you clarify this sentence?

*The scale midpoints of the seven-point rating scales have been clarified in this sentence.*

8. Line 236: here or before when describing the timing of saliva sample can you elaborate on the clearance delay of both cortisol and testosterone in saliva and give background citation to support the adequacy of your protocol?

*In this section, the authors have added information about the timing of saliva sample collection – both in terms of time of day and clearance delay, citing existing background citation used in the manuscript.*

9. Line 246:

- what does ‘base on concentration’ mean here.

- Explain here already that you used high and low QCs?

- Give CVs for each separately.

- Also why dii you calculate intra-assay CV as “an average value calculated from the individual CVs from all the plate duplicates”. You should use the average CV only from the duplicates of the QCs and not from your sample as you do not know if theyare contaminated or there were sampling error.

- Obviously the ducplicates with high CV should be removed and reanalyzed. Can you clarify if you did that and what was your cuttof for reanalysis?

*“Based on concentration” refers to the fact that CV values were calculated from concentration rather than raw optical density. The explanation of high and low controls being used is included in line 274 of the manuscript.* *Next to the* *average inter-assay CVs for both testosterone and cortisol, the authors have now included the breakdown for high and low controls separately. The usage of the term intra-assay CV can vary between studies. For this protocol, the authors are using intra-assay CV calculated from sample duplicates, which is recommended by the assay protocol, as well as by Schultheiss and Stanton (2009), which is both a reference in this manuscript and a reference for this specific assay protocol. The cutoff for reanalysis used was 10%; any duplicate above this cutoff were removed and reanalyzed. This information has been added to the manuscript.*

*Schultheiss, O.C., Stanton, S.J. (2009). Assessment of salivary hormones. In: Harmon-Jones, E., Beer, J.S., eds. Methods in Social Neuroscience. New York: Guilford Press.*

10. Line 263: Please specify the distribution of single and ‘in relationship’ individuals across the TSST and the control conditions.

*This distribution information has been added to the manuscript.*

11. Line 273-276: This is methods and not results. As previously mention the authors should conduct GLMM analyses, ANOVA are highly outdated. With GLMM individual could be entered as random effect to control for the repeated sampling on each individual. I will not reiterate in details my recommendations from the first review but the authors should provide a test that their full model is significantly different from a null model comprising only the control predictors and not the test predictors.

*As mentioned in the response to reviewers from the first review,* *the reviewers recommended two different statistical approaches for revisions for this manuscript. Based on concerns over power, the authors chose to split analyses and only investigate two-way interactions effects, focusing on specific research questions. Both main research questions and exploratory research questions were limited to two-way interaction effects to avoid concerns regarding test power, as recommended by Reviewer #2. The results section of the manuscript had been completely rewritten based on the new analysis. Reviewer #1 (correctly) has concerns about the outdated nature of these analyses, but concerns over power prioritized these concerns for the authors when reconducting analyses.*

12. Line 358: close the citation bracket

*This typo has been resolved in the updated manuscript.*

13. The discussion as it stands is interesting and acknowledges nicely the limitations of the study but remains relatively shallow. The discussion should loop back to the theories introduced in the introduction (challenge hypothesis and dual hormone hypothesis) and specifically state whether the results here corroborate (even if only partly) or not each hypothesis.

*This comment from the reviewer was very helpful. The authors have edited the discussion to address the theories mentioned in the introduction and discuss how the results fit in with each theoretical hypothesis, particularly in the first and third paragraphs of the discussion section.*

14. Line 292: did you ask the participants specifically how they felt the interaction went and if they had the impression to flirt?

*The authors are assuming here that the reviewer is requesting information regarding Line 392 (in the discussion), as opposed to Line 292 (listed by the reviewer), as line 392 is referencing the impression of the participants in the discussion. The authors have clarified in the discussion at this point that we did not ask participants whether they were given the impression to flirt from the research assistant’s interactions.*

**Reviewer #2:**

No requested revisions to address.
